# Secure key distribution exploiting error rate criticality for radio frequency links

**DOI:** 10.1098/rsos.230411

**Published:** 2023-10-18

**Authors:** Christopher J. Stevens, Ben Allen, Anthony K. Brown

**Affiliations:** ^1^ Department of Engineering Science, Oxford University, Oxford OX1 3PJ, UK; ^2^ Electrical and Electronic Engineering, University of Surrey, Guildford, UK; ^3^ Queen Mary, University of London, London, UK; ^4^ University of Manchester, Manchester, UK

**Keywords:** communication system security, cryptography, wireless cryptography, modulation and coding, forward error correction codes

## Abstract

We propose a method by which two radio frequency (RF) communication terminals exchange encryption keys or other data securely. This method draws on the approach developed for quantum key distribution (QKD) for detecting eavesdroppers but our method does not use any quantum properties at all. Instead, by exploiting the effects an eavesdropper has on channel stability, we explore a line-of-sight link radio in which data transfer rates are so high as to approach the Shannon limit. With very steep rises in bit error rate accompanying a small degradation of signal-to-noise limits for certain forward error correction codes, it becomes possible to infer the existence of an eavesdropper before they are able to obtain a complete key. We describe our method and analyse one possible implementation using low density parity check codes with quadrature phase shift keying modulation. The proposed technique is in principle far easier to implement than quantum-based approaches for RF and optical wireless links since the required hardware is readily available and the basic principles are well known and well understood. Finally, we show our method to have a higher key rate and spectral efficiency than those of QKD.

## 1. Introduction

Secure communications is seen as essential for future personal and commercial communication systems, particularly in light of continued increases in sophistication and frequency of cyber attacks and the prospect of quantum computers becoming able to decrypt current encryption schemes in near-real time. Quantum key distribution (QKD) has been of increasing interest to provide such secure systems [1–3]. The critical benefit of these various methods is the ability, for a pair of terminals, i.e. the transmitter (Tx) and receiver (Rx), to detect the presence and interference by an eavesdropper seeking to compromise the key being shared between the Tx and Rx. By exploiting the nature of quantum measurements, these schemes enable the detection of the eavesdropper before they can obtain any useful key information. The original suggested scheme used single quanta as the medium of data exchange, which is difficult to realize in a real system because it requires the ability to detect a single energy quanta without ambiguity, i.e. to be able to distinguish between the single wanted quanta rather than that of noise at the same frequency and with the same polarization. This requires specialist equipment operating at very low temperatures and will be subject to occasional noise quanta being indistinguishable from the wanted quanta. Those demonstrations that have been successful have made use of optical and near-infrared photons to carry the information from the Tx to the Rx, often with polarization as the coding scheme. This task is made even more difficult if one attempts to create a free-space link (as opposed to a link via optical fibres for instance), as described in [4]. The reference points out that stray light and high link attenuation accrued over longer link distances renders secure key exchange as impossible when using the BB84 protocol. Using multiple photons is proposed to overcome the link losses and detection issues, which makes it vulnerable to eavesdropping, with *decoy states* being proposed to mitigate this vulnerability. This means varying the light intensity in a random manner hence emitting multiple photons, thus the link can no longer be referred to as a *quantum link* in the strict sense. References [5,6] describe *Continuous Variable QKD* or CV-QKD as opposed to *Discrete Variable QKD* or DV-QKD described above. In contrast to DV-QKD which encodes the polarization state of a single photon, CV-QKD encodes by means of Gaussian modulation and quadrature signal states and only requires standard optical transmission technologies and homodyne detection.

Some authors have considered developing a system analogous to DV-QKD using millimetre wave photons as the quanta of exchange to overcome some of the limitations of free space optical systems, but unambiguous detection of even 100 GHz photons is a difficult task requiring cryogenic temperature receivers [7,8]. The impact of thermal background noise on such systems is significant and makes detection of the quanta challenging.

If one examines the main elements of a QKD system there are three main components.

(1) The transmitter and receiver that can emit and detect single quanta.
(2) The coding which is achieved by adjusting a property of the transmitted quanta—for instance their polarization as introduced in the original BB84 scheme described in [1].
(3) A conventional public channel which is used to exchange information about the link in order to both enable the establishment of a common encryption key and determine if there is an eavesdropper present.

The presence of the public information channel is critical to the success of the scheme by providing the means by which the Rx tells the Tx the results of the data reception and permits the Rx to identify if eavesdropping has taken place. Because of the fundamental nature of quantum information it is impossible to interfere with the link without evidence being present in the reception of the data.

In this paper, we are proposing a less difficult system to implement without invoking quantum level measurements and that can detect the presence of an eavesdropper. This relies on established information and communications theory to reliably detect the presence of an eavesdropper before it can obtain any useful key information. Hence it has the same purpose as QKD.

## 2. Scheme overview

Our scheme relies on the Shannon Capacity theorem which describes the limit of data capacity for a conventional data transfer channel [9]. Unlike QKD, it does not require the manipulation of single or even a small number of photons, making it suitable for radio frequency (RF) operation, thus opening the door for enhanced security of communication links operating at frequencies below optical and where similar techniques have been highly challenging to implement due to the significantly reduced photon energy at these frequencies compared to optical frequencies. We show that our scheme can provide security against ‘man in the middle’ and ‘stand-off’ attacks for links operating at radio frequencies in a similar manner to QKD at optical frequencies, which has been a long-standing challenge and limits use to optical frequencies. Unlike the concept proposed in [10], our system does not rely on secret spreading codes that may be compromised by an adversary, nor on adding artificial noise to obscure the data, however both of these techniques may work in cooperation with our proposed scheme. Also, unlike [11] which makes use of backscatter created by the presence of an eavesdropping device, our scheme uses the signal received by the legitimate receiver to detect such a device. We anticipate our system to be far more sensitive due to it exploiting the nonlinearity of the BER versus SNR curve when operating close to the Shannon limit and we propose a further enhancement by using machine learning (ML) methods to enable close to optimal eavesdropping detection in terms of minimizing the occurrence of false positives. Thus, we propose using ML to optimally differentiate between the BER signature caused by an eavesdropper and those relating to non-malicious link perturbations. While this added feature is not essential, it would enhance performance by reducing the false positive alarm rate and hence maximize link availability.

## 3. Bit error rates and coded signals

**Figure 1.**
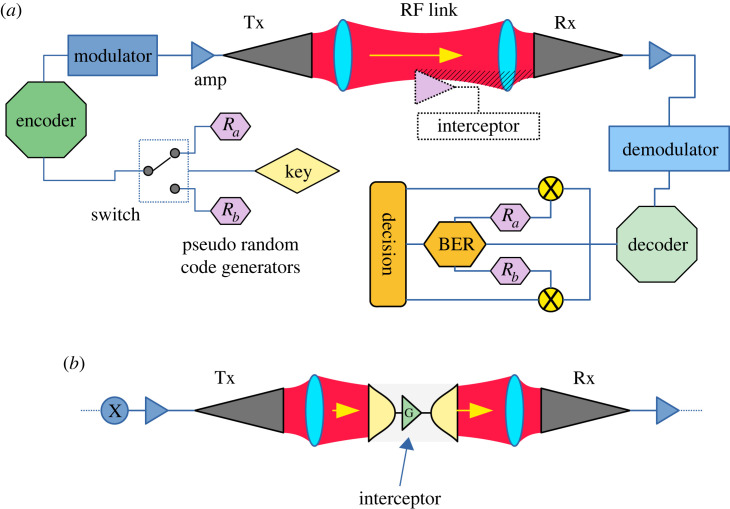
(*a*) A simple point-to-point, line of sight, highly efficient radio link where the majority of the transmitted energy arrives at the receiver with integrated means of BER-based security and including an eavesdropper. (*b*) Interception scheme via amplification and re-transmission.

Figure 1*a* shows a simple point-to-point radio link implemented using horn antennas and lenses (dishes or other high gain antennas would also suffice). Applications of such links include: mobile basestation backhaul radio links, high data rate connections where cables cannot be laid and inter-building or inter-campus data links. Such links are essential for achieving connectivity of many wide area networks which do not currently have inherent eavesdropping capability, hence making it unable to carry encryption keys. We will use this link as a model to explain our scheme.

Our method relies on the limits imposed on data transfer by thermal noise—as described by the Shannon limit for data capacity [9]. Since this is a point-to-point link, spreading path loss is negligible and operating at frequencies that incur negligible absorption loss is assumed. If we establish a link such as that shown in figure 1*a*, noise in the environment and components will limit the error performance according to the Shannon formula which compares the signal power *S* at the receiver to the total noise power received *N* to determine the ultimate data capacity *C* using the available bandwidth *B* as [9,12]

3.1 $$C=B\;\log_{2}\left(1+\frac{S}{N}\right).$$

In the figure, *R*_*a*_ and *R*_*b*_ refer to pseudo-random codes such that *R*_*a*_ is selected if the key ‘bit’ is a zero and *R*_*b*_ if the key ‘bit’ is a one.

To encode data on a link one modulates the signal after forward error correction (FEC) coding is employed to detect and correct for errors in reception. In general, no modulation and coding scheme has managed to achieve the capacity suggested by this fundamental limit but some coding schemes such as low-density parity check codes (LDPC) are beginning to approach it. Schemes such as LDPC are methods aiming to correct for transmission errors which can result in increased data capacity. For low values of SNR, they are able to reduce BER until a critically low level of SNR is reached after which error correction becomes impossible and the BER rapidly rises.

If one sets up a communication link using one of these schemes and attempts to transmit data at a rate approaching its maximum, the number of errors (bits decoded wrongly) increases until the BER approaches 0.5 and no data is decoded correctly. In effect, at these low SNRs the noise naturally occurring in the system is such that the data are unrecoverable.

**Figure 2.**
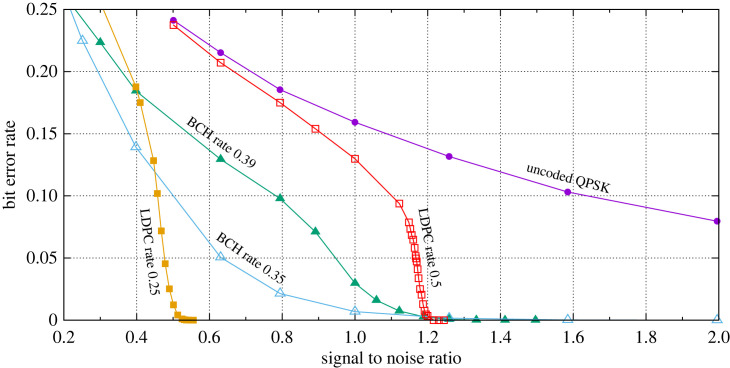
Linearly scaled graph showing various coding and modulation schemes’ bit error rate performances, against signal-to-noise ratios.

FEC coding schemes operating at a low SNR generate increasing numbers of bit errors as SNR further decreases, just the same as uncoded channels. Figure 2 shows the bit error rate for an uncoded quadrature phase shift keyed (QPSK) modulated signal compared to four examples exploiting FEC using this same modulation scheme. Two example codes are used here, Bose–Chaudhuri–Hocquenghem codes (BCH) [13] are compared to low density partiy check codes (LDPC) [14], but the scheme is not restricted to these codes. For coded data, some of the transmitted data bits are used to detect and correct the bit errors. Consequently, not all of the bits transmitted on the channel are payload data and this means that the data rate is lower than otherwise. For example, for LDPC at rate 0.5, half the bits are used for error correcting, with the other half being the data. The relative number of error correction bits controls the level of SNR at which the code can recover all the data with minimal errors. More error correcting bits makes the overall data rate lower but lowers the SNR limit required for a given BER.

Figure 2 shows the results of simulations of a communication link to investigate the BER versus SNR curves with the SNR values plotted in linear terms. These BER curves are produced by means of Monte Carlo simulation, which operates as follows.

(1) The specified SNR values are stepped through one at a time.
(2) For each step, the corresponding additive white Gaussian noise (AWGN) variance is determined to provide the required SNR.
(3) Random binary values are determined with equal 0/1 probability, which are used as the transmitted data.
(4) For the case of channel coded data, the binary data undergoes coding followed by QPSK modulation. For uncoded cases, only modulation is required.
(5) At the receiver, AWGN is then added to the received data. The signal is then demodulated and decoded.
(6) Finally, the number of bit errors in the packet is determined by comparing the received data with the transmitted data.

This process is repeated until at least 10^5^ errors have been accumulated before proceeding to the next SNR value.

For the uncoded QPSK link in figure 2, the BER is already approaching 1 in 10 (0.1) for SNR of 1.6 with a gently rising error rate as SNR further decreases. In strong contrast to this behaviour are the coded links where, on this linear scale, bit error rates stay very low, i.e. below 1 in 100 errors, until a threshold SNR (SNR_crit_) is reached after which the link rapidly degrades back to error rates comparable with the uncoded link. Of the two coding schemes considered here the steepest transition occurs for LDPC-coded signals.

## 4. A bit error rate secured link

In order to achieve security against eavesdropping, our scheme relies on the rapid increase in BER when a coding scheme is breaking down. Using the link at or very near to its SNR_crit_ means that the BER becomes very unstable with respect to decreasing SNR. By measuring the BER continuously, one can detect if anything is impeding the link and changing the SNR. All schemes for intercepting signals on the link require the eavesdropper to collect some of the transmitted link power and hence reduce the overall SNR for the link. This configuration is similar to that used by microwave beam waveguides where over 99.9% energy capture has been reported [15]. Note that the eavesdropper must also have a high enough SNR such that it can satisfactorily demodulate and decode the victim signal.

We propose to run a link such as that shown in figure 1*a* for which the SNR is set just above the critical point for the selected coding scheme where the operating BER is close to rising rapidly. In this potentially unstable condition any increasing noise or loss of signal will rapidly generate a very large number of bit errors. This is contradictory to the normal operation of such links for which the margins are generally set to maintain a required BER. In our link, we assume that all, or nearly all the power transmitted is received and that the majority of the noise power arises from the receiving electronics. We assume that the receiver is of high quality in terms of noise performance and that no significantly lower noise receiver exists—it is the best available. The consequence of this is that an eavesdropping receiver can, at best, have a comparable noise performance.

**Figure 3.**
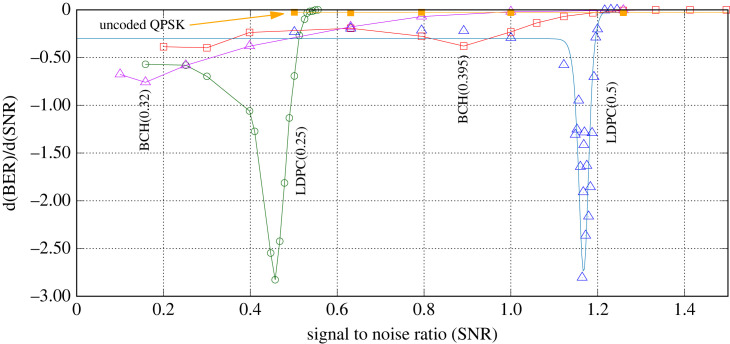
Gradient of bit error rate versus signal to noise ratio for two coding strategies, each at two different coding rates.

Figure 3 shows the respective gradients of the BER curves of figure 2. The uncoded QPSK transmission has a very modest slope whereas the two coding approaches are much stronger. Of the two, LDPC is clearly the strongest candidate with the steepest gradient. BCH has a maximum gradient of −0.75 (for rate 0.32) and −0.38 (for rate 0.39) while LDPC is −2.8 (for rate 0.5) and 2.82 (rate 0.25). The SNR value at which the maximum gradient occurs we use to quantify SNR_crit_.

Taking the LDPC case with coding rate 0.5, there is a very strong maximum negative slope of d(*BER*)/d(*SNR*) = −2.8 at SNR = 1.18. In order to maximize the link’s sensitivity to interception, we could fix the nominal SNR at this value (SNR_crit_) by adjusting the transmit power and reading the reported BER. Now using the link at this SNR, we would be expecting errors with reference to figure 2 at a rate of BER ≈ 0.05 and any increase in this value would be an indicator of interception or link occlusion and a sign of potential insecurity. In practice, this rate of errors of about 1 in 20 is inconveniently high for our link in normal operation. Instead, we can choose an operating point slightly higher than SNR_crit_ where the BER is much lower, say about 0.53 for the LDPC (0.25) case. Now, we would expect a negligible number of bit errors (BER = 3.41 × 10^-4^) for our normal operation and any reduction in the received signal will rapidly generate a rising BER as the SNR drops through SNR_crit_

Our link is thus essentially a BER test system with the transmitter sending one of two possible pseudo-random sequences (PRS). These codes need not be orthogonal but should have a low correlation to one another such that they are easily distinguished even with up to 20% of their bits in error. Note that a ‘bit’ is a ‘key bit’ when the link is used for sending an encryption key, and a key consists of a number of key bits. Similarly, a PRS consists of a number of ‘chips’. Therefore, the terms ‘bit error rate’ and ‘key error rate’ can be used interchangeably.

The receiver expects one or the other of these two PRSs and compares the arriving bit stream to a stored version of both of them, measuring the BER as it goes. Synchronization of the receiver can be achieved with a sliding correlator. Here, correlation is repeated at a range of time offsets. The time offset that produces the highest correlation indicates the synchronization point. For each of the PRSs, one will have a high maximum correlation and one a low correlation, thus allowing the receiver to identify which PRS is being transmitted at any particular time. Once the PRS is identified, bit by bit comparison delivers the BER at which it was received. The receiver will report the BER back to the transmitter by means of a *back channel*. It is this BER that is used to secure the link.

Security is provided as follows. Suppose an eavesdropper attempts to listen in on the communications, it would have to introduce a detector to achieve this. With a link running at or close to its critical bit error rate, almost all the transmitted signal power must be received in order to successfully decode the data. Merely ‘sniffing off’ a small potion of the signal energy would not be sufficient to successfully intercept and decode the data stream, however, this would reduce the signal power available at the link’s own receiver thus reducing its SNR and resulting in a dramatic increase in BER due to the critical nature of the link. This increase in error rate indicates that interception is being attempted, allowing the transmitter to identify an insecure link.

In order to provide a basis for predicting the performance of our link, we have evaluated the key error probabilities when transmitting a bit sequence across such a link. We assume that there is a memory-less channel so that each key bit transmitted has an equal probability of error *p*, then in transmitting *n* key bits we may obtain *k* errors. The probability of *k* errors in *n* key bits is then given by binomial statistics as

**Figure 4.**
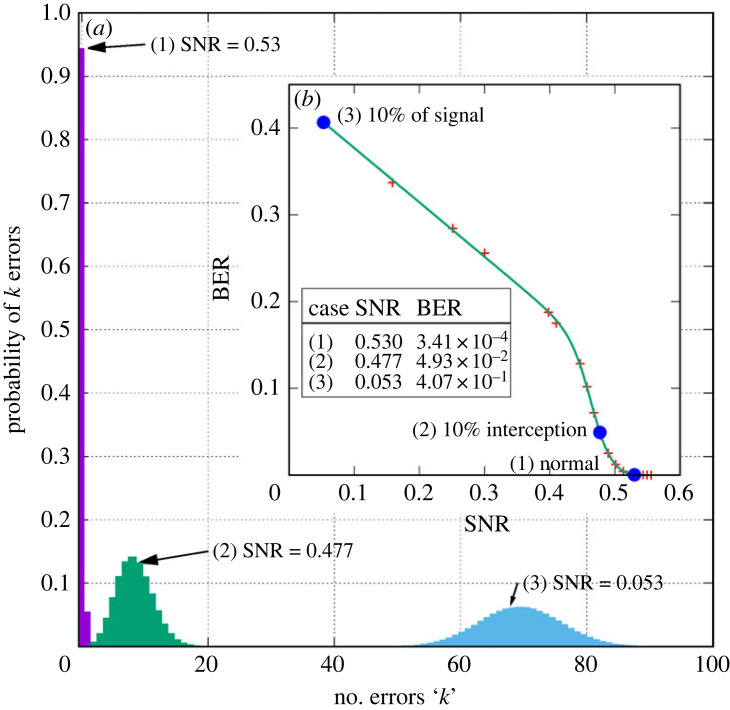
BER probabilities as SNR varies with a 10% signal power interception case, evaluated for an encryption key identified by a pseudo random sequence of 170 chips. Error statistics are obtained for the normal unperturbed link, reduced link signal power by 90% interception and for the 10% of signal power intercepted.

4.1 $$P=C_{n,k}p^{k}{\left(1-p\right)}^{n-k},$$

where *C*_(*n*,*k*)_ is the binomial coefficient *n*!/*k*!(*n* − *k*)!. Using this formula, we can test the channel behaviour as follows. Looking at the data presented in figure 2, it is possible to compare the link performance as SNR varies and we pick out three conditions for comparison listed in the inset to the figure. These are taken from the BER versus SNR results for LDPC coding at rate 0.25 which is also shown in the inset to figure 4 with the values considered shown as the three solid data points. To avoid confusion, we refer to bits of the PRS as *chips* while the bits of the key being transmitted are *key bits*. We assume that the receivers used by the link and the eavesdropper are identical and represent the best available in terms of their bandwidth and noise figure.

Using these values in equation (4.1), we can obtain results such as those shown in figure 4. Here, 170 chips are transmitted representing one bit of the key being delivered (this is the length of the PRSs used here) and the corresponding probable number of bit errors are evaluated. Figure 4 shows the error probability distribution for three different SNR operating points which are discussed in more detail in §5.1.

## 5. Eavesdropping on the link: discussion of attacks

In this section, we now consider how an eavesdropper could intercept the key from our link, and how they might be detected if they were to try.

Data on our link—the shared non-repeating encryption key to be distributed—is sent by selection of one of the two PRSs. Due to spreading using the PRS, this means we are effectively using a signalling scheme for the actual data that is well clear of the Shannon bounds and thus reliable. This data rate, *R*_*D*_, is much lower than the chip rate (*R*_*C*_) of the PRS, i.e. *R*_*C*_ ≫ *R*_*D*_. These are related by

5.1 $$R_{D}=\frac{R_{C}}{N_{c}},$$

where *N*_*c*_ is the number of chips in the PRS. Since the effective rate for the bits of the encryption key is much lower then this data could be obtainable by the eavesdropper despite their lower SNR. If the PRSs are too long then the effective key rate is so low that only a small SNR is required for successful interception—which translates as a small fraction of the total beam energy being lost. Our scheme, as described, does not prevent this interception but aims to detect it within a single key bit transmission period. The PRS length *N*_*c*_ must thus be chosen to enable reliable detection at the desired BER threshold used to detect interception, but not so long as to make it possible for that same eavesdropper to decode the key. In order to detect a particular BER, denoted as *B*_0_, one must transmit at least *N*_*c*_ ≥ 1/*B*_0_ bits so that the value chosen for the threshold BER effectively controls the length of the PRSs.

Since one bit of the encryption key being distributed is transmitted with each complete pseudo random sequence, the very rapid increase in BER enables the detection of interception within just a few bits of the key. Even if the eavesdropper can achieve a better noise floor than the main link’s receiver, they are unlikely to be able to decode the key before their interception is detected and are also receiving very much less than the optimal signal strength. Swift detection of interception also precludes the possibility of the eavesdropper gaining knowledge of which PRS represents 1’s and 0’s. To illustrate this, consider an eavesdropper with a maximum of 1/57 of the SNR relative to the main link, as stated earlier. This would require 57 times the time to receive the code, by which time the link would have shut down due to detection of the eavesdropper.

### 5.1. Man in the middle attack

Using the binomial statistics in equation (4.1), we can see what happens if an eavesdropper tries to read the key by inserting an antenna to ‘sniff off’ some of the link power. We consider a case where the link is being run a little above SNR_cirt_ at an SNR of 0.53 and the eavesdropper intercepts 10% of the link power. The results are shown in figure 4. Here, the three cases and their respective SNR and BER values are shown in the inset table.

Case (1) For the unperturbed channel where SNR is highest at 0.53, there is very low BER with 94.4% probability of zero chip errors. This would be the usual condition for this link. Users would generally see no more than one error within most key bit exchanges and all the bits of a key can be exchanged error free.

Case (2) Now an eavesdropper is trying to obtain the key by sniffing off 10% of the link power. Consequently, the eavesdropper reduces the SNR to 0.477 such that error rates rise and the probability of zero errors drops to less than 1%. Now the mean number of chip errors in each key bit rises to 8, signalling to the users that interception is likely to be occurring. It may be necessary to observe chip error rates (CER) for several key bits to establish that the eavesdropper is present, but they would certainly be detected before a complete key was transmitted.

Case (3) This is the signal the eavesdropper was receiving when intercepting 10% of the transmitted power. At this level, the eavesdropper would be unable to interpret the code transmitted having typically around 70 bit errors for each 170 bit packet. The eavesdropper would not be able to interpret key bits correctly, with each key bit being impossible to correlate with either of the PRSs employed to represent the two possible states of a key bit.

### 5.2. Amplification attack

The *amplification attack* method is shown in figure 1*b*. Here, an eavesdropping device is constructed which collects all the signal from the link’s beam, amplifies it, providing extra signal to the eavesdropper for decoding, and then re-transmitting the remaining energy along the link to avoid detection. A simple link budget calculation can be used to analyse a link where an amplifier attack is being performed. In this calculation, we assume that the link is 100% efficient in terms of energy capture and that the beamwidth at the receiver or the eaversdropper is smaller than its antenna. Now the SNR for the unperturbed link may be shown to be SNR_0_ = *S*_0_/(*N*_0_ + *N*_*r*_) where the signal power arriving is *S*_0_ and the noise powers from the transmitter and receiver are *N*_0_ and *N*_*r*_ respectively. Adding the interceptor to the link and requiring that they adjust their amplifier gain and the fraction of power that they keep so that the links’ legitimate receiver still has the same arriving signal power the SNR becomes SNR_X_ = *G*_*i*_*S*_0_/(*G*_*i*_(*N*_0_ + *N*_*r*_) + *G*_*r*_*N*_*r*_), where *G*_*i*_ and *G*_*r*_ are the gains of the interceptor and receiver amplifiers, respectively. Hence the amplifier attack has raised the SNR by a factor of

5.2 $$R_{\mathrm{amp}}=\frac{\mathrm{SN}R_{X}}{\mathrm{SN}R_{0}}=\frac{G_{i}(N_{0}+N_{r})}{G_{i}(N_{0}+N_{i})+G_{r}N_{r}}.$$

Here, *N*_0_ is the noise in the link arising from the transmitter *plus* any environmental noise that is incident on the receiving antennas. Once again, we assume that both the legitimate receiver and the eavesdropping receiver have identical capabilities with identical gains (*G*_*r*_ = *G*_*i*_) and noise levels (*N*_*r*_ = *N*_*i*_). Equation (5.2) shows that *R*_amp_ is always less than 1. In general, unless the channel and transmitter noise *N*_0_ is dominant then *R*_amp_ will always be considerably below 1 with a limiting value of 0.5 when *N*_0_ → 0.

**Figure 5.**
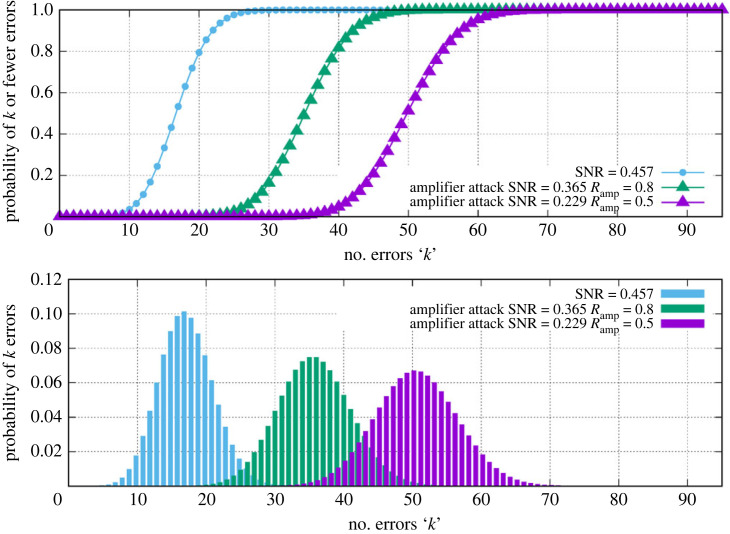
Chip error probability distributions for the unperturbed link and for amplifier attacks using *R*_amp_ = 0.8 and *R*_amp_ = 0.5.

Figure 5 shows the statistics for this attack. Here, the eavesdropper is calculated for two values of *R*_amp_ = 0.5, 0.8. This link is being run at SNR_crit_ = 0.457 with LDPC at rate 0.25 as before. The normal channel exhibits a mean error rate of 16 chip errors per key bit. Even with the higher value of *R*_amp_ = 0.8 the perturbed channel is quite obvious from the noise statistics with a mean that rises to 36 chip errors per key bit. Thus, although the amplifier attack enables the key to be intercepted by the eavesdropper, it is immediately obvious that this interception is taking place before the whole key has been transmitted.

### 5.3. Regenerative attacks

A more complex attack can be made by *regenerative* interception where an eavesdropper receives the entire signal, demodulates it, then uses it to generate a new, identical signal at an identical power level toward the receiver—a regenerator. Now the detection of eavesdropping is potentially much harder. If the link is being run at an SNR too far above SNR_crit_ as in figure 4 then detection becomes nearly impossible. If however the link is run at or close to SNR_crit_, once again it is still reasonable to detect this kind of attack. Now one aims to run the legitimate link in such a way that there are always a detectable number of chip errors. In this case, the regenerator may reproduce the data it receives faithfully, but it cannot avoid that data already containing these errors due to its own receiver limitations being the same as ours. These can only increase in number when the user receives the regenerated signal. This is a kind of ‘bush telegraph’ effect that is unavoidable with each receiver adding noise. The presence of the regnerator essentially changes the statistics of the bit errors thus modifying equation (4.1) which becomes

**Figure 6.**
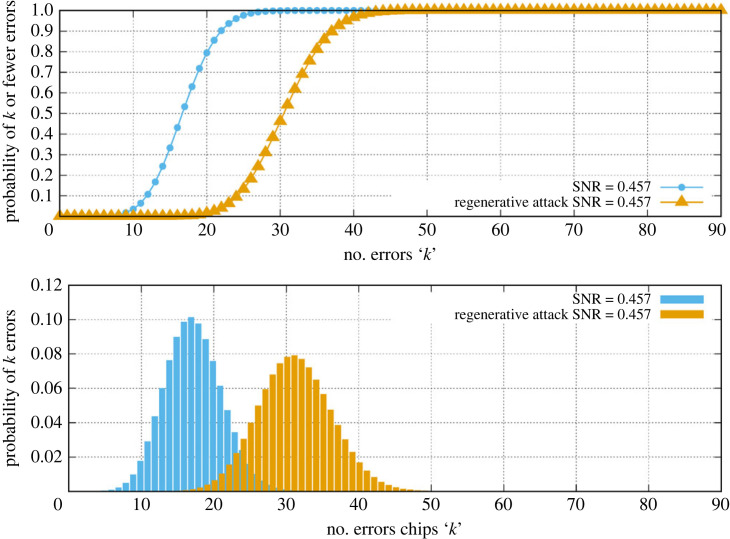
Chip error probabilities based on SNR = 0.457 with a regenerative attack mode.

5.3 $$P_{rg}(k)=C_{n,k}{\left(2p(1-p)\right)}^{k}{\left(1-2p+2p^{2}\right)}^{n-k},$$

where the parameters are the same as before. This arises because each receiver will experience chip errors, mostly in differing chips and generally increasing the overall number. Using equation (5.3), one can explore the impact on the chip error statistics for the LDPC rate 0.25 link when run at SNR_crit_. Figure 6 shows the predicted chip error statistics. Again the interception has increased chip error rates (CER) by a significant amount from a mean of 16 to 31. While this is a smaller contrast when compared with the amplifier attack at *R*_amp_ = 0.8, it is still quite a clearly detectable condition. The slightly greater overlap between the error distributions means it may be necessary to observe the channel for a number of key bits to achieve a positive detection of the regenerator.

### 5.4. Stand-off attack

So far the discussion has focused on the interception device located between the transmitter and receiver. Section 4 described the use of a focused beam system with very efficient transmission. We now consider using our method on a more conventional link and consider a *standoff attack* where the interception device is located with an angular offset away from the direct path. The use of antennas such as a parabolic reflector antenna for forming a point-to-point link as shown in figure 1*a* allows a low level of energy to spill past the receiving antenna due to the sidelobes in the antenna radiation pattern. The exact profile of energy versus angle depends on antenna design detail. We consider two examples: the first uses small reflector antennas (20 cm diameter transmitting at 3.5 GHz over a range of 10 m) which exhibit relatively high side lobe energy. The second example is for a much higher frequency and wider diameter reflector (4.6 m diameter transmitting at 42 GHz transmitting over a range of 1 km). The operating frequencies and aperture diameters were selected from the minimum and maximum values from [16]. We consider these two examples to illustrate likely practical extremes. The far-field radiation pattern may be closely approximated [17] using equation (5.4)

5.4 $$P(\phi )=P_{0}{\left[\frac{2J_{1}(\pi D\;\sin(\phi )/\lambda )}{\left(\pi D\;\sin(\phi )/\lambda \right)}\right]}^{2},$$

where *D* is the diameter of the reflector, *P*_0_ is the launched power, *λ* is the wavelength of the signal, *J*_1_(*x*) is a bessel function of the first kind and *ϕ* is the angular offset from the beam centre.

**Figure 7.**
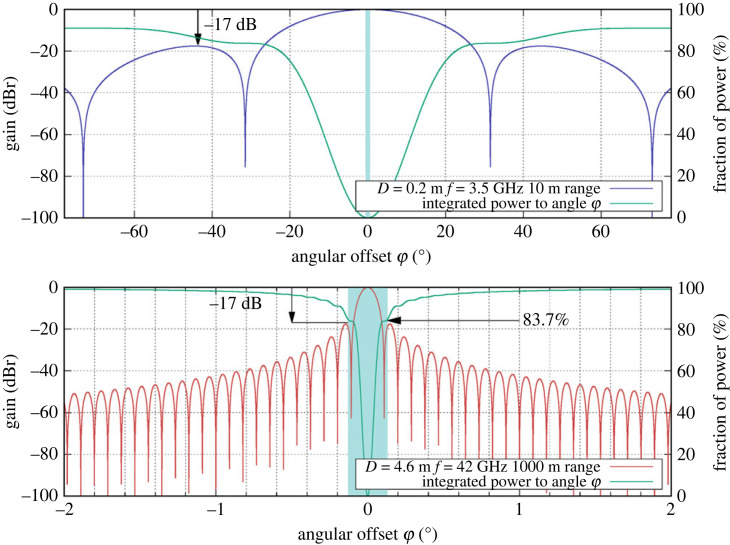
Relative gain versus angle for two centre fed parabolic reflector antennas at two different ranges. Shaded regions correspond to angles subtended by corresponding receiver dishes where the 3.2 GHz link has only 10 m range and the 42 GHz link has 1 km range.

Figure 7 shows the beam patterns for these two extreme cases where the arrows mark the highest levels of power outside of the main beam which are both −17 dB. In both cases, a matching identical dish is assumed as the receiver. The smaller 0.2 m diameter dish, even though it is being operated at short range of only 10 m, has the majority of its radiated power outside of the receiver’s footprint with only 0.13% actually being received. The larger 4.6 m diameter dish with much shorter wavelength signals generates a much tighter beam where more than 83% of the radiated energy is captured by the matching receiver dish.

In the first case, even at short range, our system would fail due the majority of the signal power missing the receiver and being available to a standoff eavesdropper. The second case however is much better. Now a standoff eavesdropper can, at best, acquire only 16.3% of the signal energy and only then by collecting all the sidelobe energy. We would design our PRS pair so that this level of error in the eavesdropper’s data renders reliable decoding impossible.

**Figure 8.**
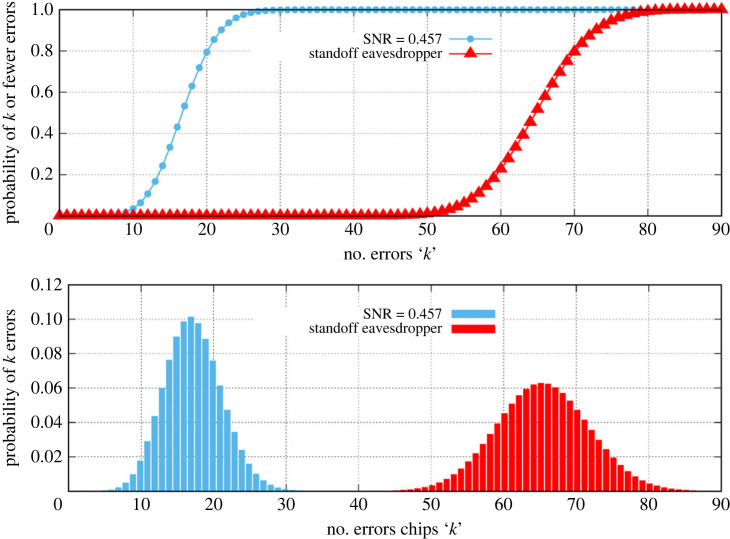
Chip error probabilities for the direct link at 42 GHz, over 1000 m using 4.6 m dishes, and for a standoff eavesdropper that captures all of the available stray power.

We assume that the link is again adjusted so that its receiver is operating with an SNR of 0.457. The chip-error analysis for the 42 GHz link using 4.6 m dishes is shown in figure 8 where the eavesdropper is assumed to be able to collect all of the lost signal energy by some Herculean effort of antenna construction. While the link operators would not observe any change in the link statistics to identify this attack, it would still fail. Even with this huge eavesdropping receiver antenna, the eavesdropper has to contend with on the order of 65 chip errors per key bit making the key impossible to decode reliably. The link will only transmit the key once so an attacker may not rely on signal averaging or other methods to acquire the full key.

This situation can be made even more secure by raising the frequency and narrowing the beam to further reduce the fraction of lost energy, with operation at optical frequencies being attractive but without requiring the complexity of traditional QKD techniques.

## 6. Eavesdropper detection

Having considered four different eavesdropping scenarios, it is clear that a continuous monitoring of the chip error rate is sufficient to identify when any of these attacks are being made apart from the stand off attack (which is rendered hopeless by good antenna design in any case). No simple interception can succeed and attacks based on amplifiers and regenerators are quite straightforward to detect as long as the performance of all receivers used is comparable. In general, the continuous reporting of the chip error rate provides for this detection.

**Figure 9.**
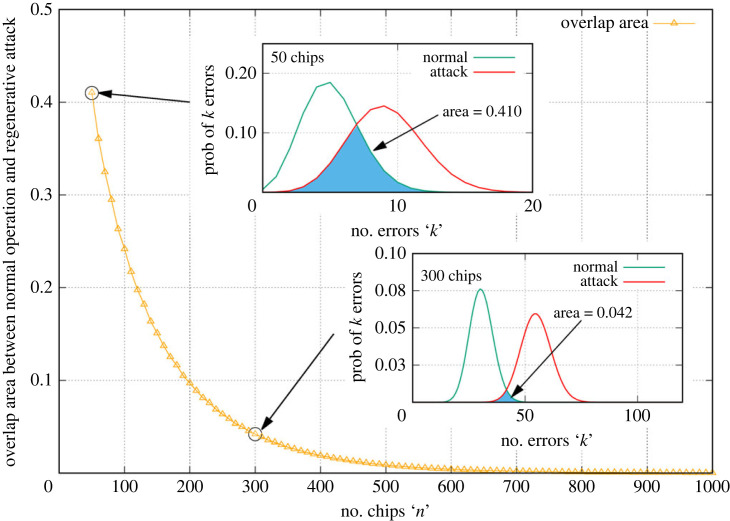
Adjusting the number of chips per key bit to minimize overlap and facilitate trivial eavesdropper detection.

Figure 9 shows how one can ensure that the detection is robust. The degree of overlap between the CER distributions can be continuously adjusted by changing the number of chip bits in the PRS making up a key bit. The overlapping area decreases exponentially with increasing chip number, with a factor of 10 improvement between 50 and 300 chips. The length of PRS used for each of the key bits should then in general be the longest that can be used while still maintaining the minimum required key rate.

A remaining question is ‘how can the perturbation in CER be attributed to an eavesdropper and not something else?’. This refers to the decision block shown in figure 1*a* where a decision is made whether to indicate to the transmitter to cease transmission of the key. Our approach uses CER as a way to sensitively determine changes in signal-to-noise ratio from the link. The error statistics of the wanted transmitted signal are under the control of the system. This allows us to choose our wanted signal to exhibit very different error statistics compared with changes in CER caused by other events. For example, for a point-to-point link, the CER would be set up to exhibit a constant level. With an interception device subsequently inserted in the link, the CER would rapidly increase thus indicating an event and that action should be taken.

Avoiding excessive false alarms is important to system performance, and thus selecting an optimum decision threshold based on comparison between the measured CER signature and stored signatures is required [18,19]. One such ML method, for instance, is by training a neural network with CER signatures relating to a wide range of items and geometries that might occlude the link such as birds or weather events; as well as items that would resemble an eavesdropper's antenna. Extensive laboratory testing to identify a wide range of link perturbations is required to train the neural network prior to operational deployment. Signatures would be collected for a wide range of non-malicious perturbations as well as signatures synthesizing a malicious eavesdropper. Due to differences in materials, geometries and temporal behaviour, we can expect non-malicious perturbations to differ from those of a malicious eavesdropper. For example, an eavesdropper would consist of a metallic structure (antenna) of a certain size and geometry inserted into the link in a preferred way hence creating a related CER signature. The learning process would only need to be done once and the configuration copied to all deployed units, along with updates being issued during operation. These stored signatures are then compared with those obtained during operation and used to determine whether a CER signature should be attributed to an eavesdropper.

## 7. Comparison with QKD

References [20,21] each describe an experimental free space optical QKD system with the performance shown in table 1. The table also shows the parameters for our proposed system where the following assumptions and observations have been made.

(1) **SNR.** This is set to 0.457 which is the critical SNR for an LDPC code with rate 0.25, see figure 3.
(2) **BER.** With reference to figure 2, an SNR of 0.457 corresponds to a BER of 0.119 for the LDPC code with rate 0.25.
(3) **Noise bandwidth.** This is assumed to be 160 MHz to be consistent with that of cutting-edge microwave point-to-point communications products [22]. We anticipate this bandwidth to increase as technology evolves, for example for equipment operating in the millimetre wave bands.
(4) **Spreading factor.** This is the number of chips in the PRS that make up a single key bit and are termed *N*_*c*_. Since the BER is approximately 10% in this example, we require *N*_*c*_ > 20. The larger *N*_*c*_ is, the higher the confidence of determining BER accurately at the receiver. However, the larger *N*_*c*_ becomes, the lower the key rate will be. We select *N*_*c*_ = 200 for this example.

We can determine the key rate using the Shannon capacity theorem provided in equation (3.1) as follows.

7.1 $$C=160\times 10^{6}\;\log_{2}(1+0.457)R_{c}/N_{c}.$$

Hence, using the parameters above gives *C* = 108 kb s^−1^ which is substantially greater than the two other examples, as shown in the table. Furthermore, while the key error rate is higher than the other two examples, a higher SNR can be selected. A very slight increase in SNR by 0.1 to 0.557 results in a BER of less than 3 × 10^−6^. Using this value would result in a probability of zero errors *p*_zero_ > 0.999. This would make this link more efficient and also reduce the false positive rate, however the trade-off would be a slight reduction in sensitivity.

**Table 1.** Performance parameter comparison between two QKD systems and our system.

system	FEC	noise bandwidth	key rate	key error rate	spectral efficiency b/s/Hz
QKD1 [20]	**LDPC**	6 × 10^6^ THz	20−400 b s^−1^ (depending on atmospheric variations)	0.039 and 0.0918 (for each of 2 states)	3.3 × 10^−18^ to −6.7 × 10^−17^
QKD2 [21]	**Turbo**	30 × 10^3^ THz	500 b/s	0.034	5 × 10^−15^
our system (SNR = 0.457)	**LDPC**	160 MHz	108.6 kb s^−1^	0.119	6.8 × 10^−4^
our system (SNR = 0.53)	**LDPC**	160 MHz	128.6 kb s^−1^	3 × 10^−6^	8 × 10^−4^

It is clear from table 1 that our system with SNR = 0.557 provides the highest key rate, lowest key error rate and highest spectral efficiency of the candidate systems.

## 8. Conclusion

Communication links using classical methods are limited by the signal-to-noise ratio for a given level of error performance. At a critical value of this ratio, we find that the rate of incorrect bit transmission can change very quickly. Using the extremely rapid change in bit error rate provided by an LDPC code with a rate of 0.25 for signals at around SNR = 0.457, we have found that the BER itself can be used as a method for detecting any attempts to intercept an efficient line-of-sight radio link. Creating a link in which the SNR is carefully gauged to be close to the point of maximum BER slope enables very sensitive detection of signal eavesdropping. The receiving terminal need only report their measurement of bit error rate back to the transmitter to ensure the link quality. A shared encryption key may be transmitted across such a link using a pair of PRS with which to encode the encryption key data without concern that it may be intercepted without detection. Fundamental communications theory (SNR requirements as expressed in Shannon’s channel capacity theorem) prevents a simple eavesdropper from successfully intercepting the signals while alerting the users to the attempt. Our method, while discussed here in terms of radio links, would be equally applicable to optical links as they are also subject to the same limits.

While the scheme presented here is suited for point-to-point links, it may be integrated with directional modulation such as the scheme reported in [23]. The two schemes would compliment each other in that our scheme provides protection from on-axis eavesdropping, while directional modulation provides protection from off-axis eavesdropping, making a highly secure radio link through reducing the probability of interception while increasing the probability of detection an eavesdropper. Our scheme may also be applied to protecting wire, waveguide or fibre communication channels as these can be considered as point-to-point.

We believe our paper to be the first paper describing this technique.

## Data Availability

Please see electronic supplementary material. The final paper will have the following text: Data from the simulations used to produce the results in figures 2 and 3 are available from: https://doi.org/10.6084/m9.figshare.c.6895258 [24].
